# Solubility of gaseous hydrocarbons in ionic liquids using equations of state and machine learning approaches

**DOI:** 10.1038/s41598-022-17983-6

**Published:** 2022-08-22

**Authors:** Reza Nakhaei-Kohani, Saeid Atashrouz, Fahimeh Hadavimoghaddam, Ali Bostani, Abdolhossein Hemmati-Sarapardeh, Ahmad Mohaddespour

**Affiliations:** 1 Department of Chemical and Petroleum Engineering, Shiraz University, Shiraz, Iran; 2 Department of Chemical Engineering, Amirkabir University of Technology (Tehran Polytechnic), Tehran, Iran; 3 Key Laboratory of Continental Shale Hydrocarbon Accumulation and Efficient Development (Northeast Petroleum University), Ministry of Education, Northeast Petroleum University, Daqing, 163318 Heilongjiang China; 4 Institute of Unconventional Oil and Gas, Northeast Petroleum University, Daqing, 163318 China; 5 College of Engineering and Applied Sciences, American University of Kuwait, AUK, P.O. Box 3323, Salmiya, Kuwait; 6 Department of Petroleum Engineering, Shahid Bahonar University of Kerman, Kerman, Iran; 7 Department of Chemical Engineering, McGill University, Montreal, QC H3A 0C5 Canada

**Keywords:** Chemistry, Engineering, Mathematics and computing

## Abstract

Ionic liquids (ILs) have emerged as suitable options for gas storage applications over the past decade. Consequently, accurate prediction of gas solubility in ILs is crucial for their application in the industry. In this study, four intelligent techniques including Extreme Learning Machine (ELM), Deep Belief Network (DBN), Multivariate Adaptive Regression Splines (MARS), and Boosting-Support Vector Regression (Boost-SVR) have been proposed to estimate the solubility of some gaseous hydrocarbons in ILs based on two distinct methods. In the first method, the thermodynamic properties of hydrocarbons and ILs were used as input parameters, while in the second method, the chemical structure of ILs and hydrocarbons along with temperature and pressure were used. The results show that in the first method, the DBN model with root mean square error (RMSE) and coefficient of determination (R^2^) values of 0.0054 and 0.9961, respectively, and in the second method, the DBN model with RMSE and R^2^ values of 0.0065 and 0.9943, respectively, have the most accurate predictions. To evaluate the performance of intelligent models, the obtained results were compared with previous studies and equations of the state including Peng–Robinson (PR), Soave–Redlich–Kwong (SRK), Redlich–Kwong (RK), and Zudkevitch–Joffe (ZJ). Findings show that intelligent models have high accuracy compared to equations of state. Finally, the investigation of the effect of different factors such as alkyl chain length, type of anion and cation, pressure, temperature, and type of hydrocarbon on the solubility of gaseous hydrocarbons in ILs shows that pressure and temperature have a direct and inverse effect on increasing the solubility of gaseous hydrocarbons in ILs, respectively. Also, the evaluation of the effect of hydrocarbon type shows that increasing the molecular weight of hydrocarbons increases the solubility of gaseous hydrocarbons in ILs.

## Introduction

Ionic liquids (ILs), unlike most salts, are substances that are liquid at temperatures lower than 373.15 K. As a result of various combinations of cations and anions, numerous ILs with tunable properties can be obtained^1,2^. ILs have become commonly used in different industries due to their various appealing properties, such as thermal stability, non-flammability, and various electrical and ionic conductivity. Therefore, many ILs have been synthesized for various purposes in recent decades^3,4^. The implementation of effective solvents is critical in the battle to conserve the environment and eliminate dangerous atmospheric concentrations^5,6^. ILs are one of the most prominent eco-friendly solvents. ILs might be regarded as attractive compounds with various potential industrial uses because of these exceptional properties^5–7^. Recent works on ILs have concentrated on structure–property correlations to intelligently develop ILs for special purposes. The structure–property interactions related to gas solubility in IL are of considerable importance. The application of ILs in separation processes highlights the relevance of gas solubility in ILs. Acknowledging the dissolution process of substances in ILs and how they might investigate the key structural properties of the solvent seems to be a more basic objective^8,9^. For many purposes, employing ILs to absorb the major components of natural gas has been a popular study subject. Primarily, such information is required to investigate the natural gas sweetening operation, which involves the separation of acid gases from methane. This technique is important not only for ecological reasons, but also for preventing subsequent process issues including equipment corrosion and pipeline clogging^5,10^. Furthermore, such information is useful for a variety of operations, including supercritical fluid extraction and homogeneous interactions in ILs, as well as purification of steam converting or gas-shift processes^3,5^. Since ILs are structurally heterogeneous on a very small scale, the solubility of substances may differ from that of molecular liquids. The solubility of pure gaseous hydrocarbons is also a promising topic for investigating the performance of nonpolar component solvation in the ILs. While some experimental researches of gaseous hydrocarbons solubility in ILs have been conducted, the majority of these researches have relied on gas solubility in ILs based on thermodynamic properties^8^.

Therefore, the study of the solubility of various gases, especially gaseous hydrocarbons in ILs is of special importance. There are various methods such as laboratory tests and the employment of equations of state to measure the solubility of gaseous hydrocarbons in ILs. Many studies have been performed on the solubility of methane^5,11^, ethane^12,13^, normal butane and isobutene^8^, and other gaseous hydrocarbons in various ILs. Ahsan Jalal^14^ calculated the solubilities of C4-hydrocarbons in various imidazolium-type ILs using the Conductor-like Screening Model for Realistic Solvents (COSMO-RS) computations. The COSMO-RS data for each hydrocarbon was then examined utilizing semi-empirically determined molecular descriptors of ILs and machine learning technologies such as association rule mining and decision tree categorization. The most essential characteristics for defining the propensity of ILs towards C4-hydrocarbons are the polarizabilities of both cation and anion, as well as the anion's CPK (space-filling model) area. Zhen Song^15^ used COSMO-RS to forecast the solubilities of 220 imidazolium-based ILs in seven fuel hydrocarbon samples with varying IL and hydrocarbon properties. The nitrogen analyzer was used to measure the solubilities of five typical ILs in the examined hydrocarbons, revealing COSMO-RS's adequate capacity to forecast IL-in-hydrocarbon solubilities. AlSaleem^16^ investigated the solubility of three halogenated hydrocarbons in 12 hydrophobic ILs at temperatures ranging from 25 to 45 °C. Piperidinium, pyrrolidinium, and ammonium-based cations coupled with [TF_2_N] anion were studied for their chemical structure and alkyl chain length impact. Carbon tetrachloride and bromoform are somewhat miscible in all of the ILs tested, although chloroform is fully miscible. The solubility of ammonium-based ionic liquids rises as the cation molecular weight and alkyl chain length increase. Hamedi et al.^5^ calculated the solubility of methane in ILs using two equations of state including modified Sanchez and Lacombe (SL) and cubic-plus-association (CPA). Comparison of the results showed that the mentioned methods have good accuracy in predicting the solubility of methane in ILs. Owing to the very non-ideal reactions, thermodynamic analysis of gas solubility in ILs is a difficult undertaking. Typical EOSs, on the other hand, overlook the influence of several essential non-ideal molecular interactions, like hydrogen bonds and polar interactions, and so are unreliable in predicting gas solubility in ILs^5,17^. This demands the evaluation of more powerful EOSs, which are capable of adequately modeling the system across a broad temperature and pressure range and have reasonable conformance with the real performance of the system. Recently, machine learning-based approaches have widely been received attention for predicting properties of ILs^18–24^. The excellent results of previous studies further demonstrate the reliability and applicability of these methods. Other advantages of these methods are their high accuracy in predicting the solubility of gases in ILs and reducing time and cost. Ferreira et al.^25^ conducted a study on binary systems of ionic liquids and liquid hydrocarbons and their modeling by COSMO-RS. The results showed that COSMO-RS can be a useful and practical predictive tool. The employment of intelligent methods to predict the solubility of various gases such as N_2_O^26^, SO_2_^27,28^, H_2_S^24,29^, and CO_2_^30^ in ILs has received much attention in the last decade, and the results confirm the reliability of these methods. Using the thermodynamic properties of ILs and 728 experimental data, Baghban et al.^31^ developed a multi-layer perceptron (MLP) model and an adaptive neuro-fuzzy interference system (ANFIS) to predict the solubility of CO_2_ in ILs and then compared the results with the equations of state. The results showed that the MLP model had the best performance with mean square error (MSE) and coefficient of determination (R^2^) values of 0.000133 and 0.9972, respectively. Ahmadi et al.^32^ did the same work by developing artificial neural networks optimized with backpropagation (BP) and particle swarm optimization (PSO) algorithms to predict the solubility of H_2_S in ILs. The mean square error (MSE) and R^2^ values for PSO-ANN were 0.00025 and 0.99218, respectively, which indicated the better performance of this model than BP-ANN. Song et al.^33^ predicted the solubility of CO_2_ in various ILs by using a very large database containing 10,116 experimental data points. In this study, artificial neural networks and support vector machine (SVM) were used to develop the group contribution (GC) method. The results showed that the ANN-GC model had the best performance with MSE and R^2^ values of 0.0202 and 0.9836, respectively. In 2021, Moosanezhad-Kermani et al.^34^ developed a group method of data handling method for prediction CO_2_ solubility in ILs. In 2022, Mohammadi et al.^35^ predicted SO_2_ solubility in ILs using four intelligent models and equations of state.

In this study, using an extensive data bank including 2145 experimental data points, four intelligent models including Extreme Learning Machine (ELM), Deep Belief Network (DBN), Multivariate Adaptive Regression Splines (MARS), and Boosting-Support Vector Regression (B-SVR) are developed to predict the solubility of gaseous hydrocarbons (CH_4_, C_2_H_6_, C_3_H_8_, C_4_H_10_, C_2_H_4_, C_3_H_6_, C_4_H_8_, and C_6_H_6_) in ILs in a wide range of temperature and pressure. In this regard, intelligent models are trained based on two strategies, including Model (I): based on the thermodynamic properties of ILs and hydrocarbons, and Model (II): based on the chemical structure of ILs and hydrocarbons. In the first method, the critical properties of ILs and hydrocarbons and their molecular weights are considered as input parameters to the model. In the second method, the effect of the substructures that make up ILs and gaseous hydrocarbons are considered. In the development of existing models, the data is divided into an 80/20 ratio for the training and testing stages. Results are compared with previous studies and equations of state to evaluate the precision of the developed model. Furthermore, the solubility trend study is also performed to investigate the effect of different parameters on the solubility of gaseous hydrocarbons in ILs.

### Data collection

**Figure 1:**
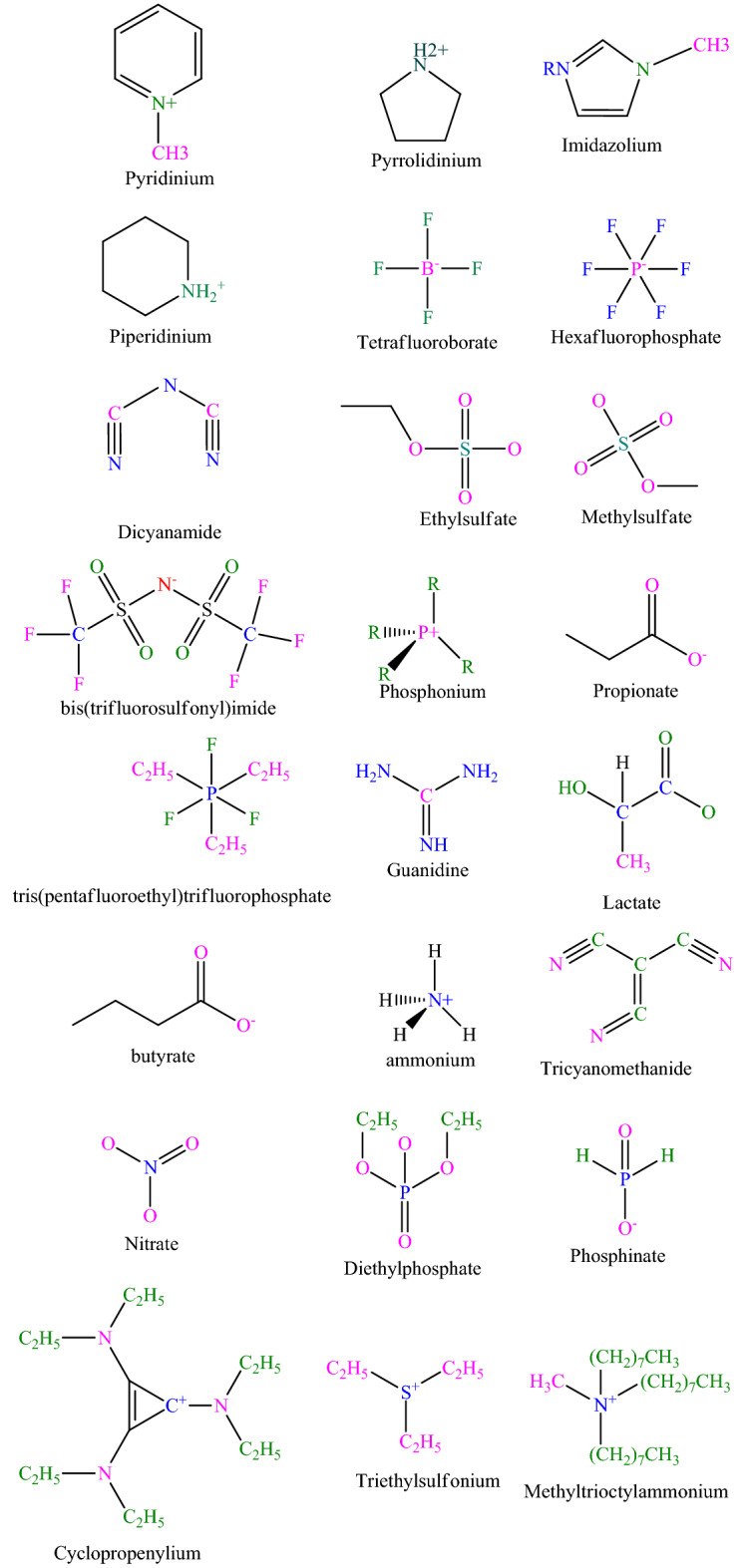
Chemical structure of some of anions and cations studied in this work.

In this study, 2145 experimental data for the solubility of gaseous hydrocarbons (CH_4_, C_2_H_6_, C_3_H_8_, C_4_H_10_, C_2_H_4_, C_3_H_6_, C_4_H_8_, and C_6_H_6_) in ionic liquids over a wide range of temperature (280–453.15 K) and pressure (0–201.64 bar) have been collected from the literature^3,4,12,13,36–54^. The details of the provided database are summarized in Table 1. In the method based on thermodynamic properties, temperature, pressure, molecular weight, boiling point, and critical properties of ILs and hydrocarbons are considered as input parameters, while in the method based on chemical structure, in addition to the substructures of ILs and hydrocarbons, temperature and pressure are considered as input parameters. The statistical description of the input parameters and a number of substructures used in this study are reported in Table 2 and Table 3, respectively. The data are then divided into an 80/20 ratio in the training and testing stages, respectively. Figure 1 illustrates the chemical structure of anions and cations used in this study.

**Table 1:** Description of systems of ionic liquids and hydrocarbons used in this study.

IL full name	IL abbreviation	Hydrocarbon	T (K)	P (bar)	S (mole fraction)	N
1-Butyl-3-methylimidazolium tetrafluoroborate	[BMIM][BF_4_]	CH_4_	283.05–343.09	0.465–0.976	0.00045–0.00126	13
1-Ethyl-3-methylimidazolium tris(pentafluoroethyl)trifluorophosphate	[EMIM][eFAP]	CH_4_	293.3–363.42	20.76–86.92	0.052–0.155	31
1-Ethyl-3-methylimidazolium ethylsulfate	[EMIM][EtSO_4_]	CH_4_	292.31–293.63	1.98–101.5	0.0013–0.0405	8
1-Butyl-3-methylimidazolium methylsulfate	[BMIM][MeSO_4_]	CH_4_	293.15–413.2	13.63–88.53	0.0091–0.046	24
1-Butyl-3-methylimidazolium hexafluorophosphate	[BMIM][PF_6_]	CH_4_	283.15–343.12	0.04–13.99	0–0.1138	99
1-Butyl-3-methylimidazolium bis(trifluoromethylsulfonyl)imide	[BMIM][Tf_2_N]	CH_4_	300.13–453.15	15.01–161.05	0.0298–0.2245	124
1-Hexyl-3-methylimidazolium bis(trifluoromethylsufonyl)imide	[HMIM][Tf_2_N]	CH_4_	293.3–413.25	0.0158–93	0.0007–0.186	125
1-Hexyl-3-methylpyridinium bis(trifluoromethylsufonyl) imide	[HMPY][Tf_2_N]	CH_4_	298.15–333.15	0.0158–10	0.0007–0.0277	50
1,1,3,3-tetramethylguanidine lactate	[TMG][L]	CH_4_	308–328	10.4–103.4	0.0009–0.0431	30
1-Hexyl-3-methylimidazolium tricyanomethanide	[HMIM][TCM]	CH_4_	293.26–363.37	18–103.6	0.025–0.1	31
N-Methyl-(2-hydroxyethyl)amine propionate	[m2HEA][Pr]	CH_4_	313–363.1	25–153.9	0.0153–0.0784	44
Bis(2-hydroxyethyl)amine propionate	[BHEA][Pr]	CH_4_	333.1–363.1	48.2–114.6	0.0141–0.0537	16
(2-hydroxyethyl)amine propionate	[2HEA][Pr]	CH_4_	333.1–363.1	48.2–114.6	0.0141–0.0537	16
Bis(2-hydroxyethyl) ammonium butanoate	[BHEA][Bu]	CH_4_	313–353	24.26–201.64	0.018–0.073	20
1-Hexyl-3-methylimidazolium nitrate	[HMIM][NO_3_]	CH_4_	293.15–343.15	8.74–30.55	0.0204–0.0993	30
1-Ethyl-3-methylimidazolium diethylphosphate	[EMIM][dep]	CH_4_	303.17–363.29	16.85–94.41	0.02–0.076	35
Trihexyltetradecylphosphonium bis(2,4,4-trimethylpentyl)phosphinate	[thtdp][phos]	CH_4_	302–363.27	10.15–120.49	0.107–0.496	42
Trihexyltetradecylphosphonium dicyanamide	[thtdp][dca]	CH_4_	302.13–363.48	14.28–115.69	0.079–0.343	49
1-Allyl-3-methylimidazolium dicyanamide	[amim][dca]	CH_4_	303.19–363.68	33.51–95.9	0.015–0.034	28
1-Butyl-1-methylpyrrolidinium dicyanamide	[bmpyrr][dca]	CH_4_	303.22–363.66	25.59–68.98	0.019–0.041	28
1,2,3-Tris(diethylamino)cyclopropenylium dicyanamide	[cprop][dca]	CH_4_	303.23–363.78	18.93–70.55	0.029–0.086	28
1,2,3-Tris(diethylamino)cyclopropenylium bis(trifluoromethylsulfonyl)imide	[cprop][Tf_2_N]	CH_4_	302.82–363.42	18.8–78.85	0.066–0.19	35
1-Butyl-1-methylpiperidinium bis(trifluoromethylsulfonyl) imide	[bmpip][Tf_2_N]	CH_4_	303.03–363.66	14.11–73.83	0.033–0.12	35
Triethylsulfonium bis(trifluoromethylsulfonyl)imide	[tes][Tf_2_N]	CH_4_	303.1–363.46	12.46–82.3	0.024–0.111	35
Methyltrioctylammonium bis(trifluoromethylsulfonyl) imide	[toa][Tf_2_N]	CH_4_	302.96–363.72	11.65–75.43	0.076–0.29	35
1-Hexyl-3-methylpyridinium bis(trifluoromethylsufonyl) imide	[HMPY][Tf_2_N]	CH_4_	298.15–333.15	0.0158–10	0.0007–0.0277	50
1-Butyl-3-methylimidazolium tetrafluoroborate	[BMIM[BF_4_]	C_2_H_6_	283.02–343.22	0.423–0.936	0.00198–0.00388	12
1-Butyl-3-methylimidazolium tris(pentafluoroethyl)trifluorophosphate	[BMIM][eFAP]	C_2_H_6_	303.26–343.33	0.9437–1.083	0.0102–0.0173	15
1-Butyl-3-methylimidazolium hexafluorophosphate	[BMIM][PF_6_]	C_2_H_6_	283.15–343.12	0.0002–13	0–0.043	172
1-Butyl-3-methylimidazolium bis(trifluoromethylsulfonyl)imide	[BMIM][Tf_2_N]	C_2_H_6_	283.15–323.15	0.000425–13	0–0.126	70
1-Butyl-1-methylpyrrolidinium tris(pentafluoroethyl)trifluorophosphate	[BMPYR][eFAP]	C_2_H_6_	303.16–344.69	0.3887–0.92	0.00573–0.013	12
1-Ethyl-3-methylimidazolium ethylsulfate	[EMIM][EtSO_4_]	C_2_H_6_	322.76–349.98	2.1–40.06	0.0016–0.0369	13
1-Ethyl-3-methylimidazolium bis(trifluoromethylsulfonyl)imide	[EMIM][Tf_2_N]	C_2_H_6_	304.14–344.7	0.4422–0.4977	0.0038–0.0066	9
1-Hexyl-3-methylimidazolium tris(pentafluoroethyl)trifluorophosphate	[HMIM][eFAP]	C_2_H_6_	303.14–343.14	0.5204–0.6656	0.0091–0.0167	13
1-Hexyl-3-methylimidazolium bis(trifluoromethylsufonyl)imide	[HMIM][Tf_2_N]	C_2_H_6_	283.32–368.4	0.4404–130.7	0.0085–0.4016	118
1-Hexyl-3-methylpyridinium bis(trifluoromethylsufonyl)imide	[HMPY][Tf_2_N]	C_2_H_6_	298.15–333.15	0.0138–10	0.00076–0.132	54
Hydroxyethyl-propyl-dimethylammonium bis[(trifluoromethyl)sulfonyl]imide	[N1,1,3,2-OH][Tf_2_N]	C_2_H_6_	304.15–344.74	0.4363–0.4905	0.0036–0.0062	9
Trihexyl(tetradecyl) phosphonium tris(pentafluoroethyl) trifluorophosphate	[P6,6,6,14][eFAP]	C_2_H_6_	303.16–343.29	0.6838–0.8019	0.0253–0.0414	15
N-Pentyl-N-Methylpyrroliidiniumbis(trifluoromethylsulfonyl)imide	[pmpyrr][Tf_2_N]	C_2_H_6_	298.15–333.15	0.1–13	0.0024–0.1404	30
Diethylmethyl(2-methoxyethyl)ammonium bis(trifluoromethylsulfonyl)imide	[deme][Tf_2_N]	C_2_H_6_	298.15–333.15	0.1–13	0.0023–0.1054	30
1,2,3-Tris(diethylamino)cyclopropenylium bis(trifluoromethanesulfonyl)imide	[TDC][Tf_2_N]	C_2_H_6_	298.15–333.15	0.1–13	0.0033–0.2029	30
1-Hexyl-3-methylimidazolium tris(pentafluoroethyl)trifluorophosphate	[HMIM][eFAP]	C_2_H_6_	298.15–333.15	0.1–13	0.0034–0.195	30
1-Butyl-3-methylimidazolium tris(pentafluoroethyl)trifluorophosphate	[BMIM][eFAP]	C_2_H_6_	298.15–333.15	0.1–13	0.002–0.1499	30
1-Ethyl-3-methylimidazolium tris(pentafluoroethyl)trifluorophosphate	[EMIM][eFAP]	C_2_H_6_	298.15–333.15	0.1–13	0.0019–0.1318	30
1-Hexyl-3-methylimidazolium bis(trifluoromethylsufonyl) imide	[HMIM][Tf_2_N]	C_3_H_8_	279.98–339.97	0.999–12.182	0.0506–0.2946	33
1-Butyl-3-methylimidazolium bis(trifluoromethylsulfonyl)imide	[BMIM][Tf_2_N]	C_4_H_10_	280–340	0.21–3.511	0.0284–0.1663	16
1-Butyl-3-methylimidazolium hexafluorophosphate	[BMIM][PF_6_]	C_2_H_4_	283.15–323.15	0.01–13	0.00006–0.099	149
1-Butyl-3-methylimidazolium bis(trifluoromethylsulfonyl)imide	[BMIM][Tf_2_N]	C_2_H_4_	283.15–323.15	0.0007–13	0.0000005–0.176	57
1-Hexyl-3-methylpyridinium bis(trifluoromethylsufonyl) imide	[HMPY][Tf_2_N]	C_2_H_4_	298.15–333.15	0.0134–10	0.00057–0.146	50
1-Butyl-3-methylimidazolium bis(trifluoromethylsulfonyl)imide	[BMIM][Tf_2_N]	C_3_H_6_	279.98–339.97	0.883–10.622	0.016–0.1818	16
1-Butyl-3-methylimidazolium bis(trifluoromethylsulfonyl)imide	[BMIM][Tf_2_N]	C_4_H_8_	280–340	0.334–3.041	0.0252–0.1409	16
1-Butyl-3-methylimidazolium tetrafluoroborate	[BMIM][BF_4_]	C_6_H_6_	283.15–323.15	0.00001–0.202	0–0.43	55

**Table 2:** Statistical description of databank used in the current study.

Property	Unit	Statistical parameters					
		Mean	Median	Kurtosis	Skewness	Min	Max
T	K	321.26	318	3.58	1.44	279.98	453.15
P	Bar	24.31	9.96	3.32	1.81	1E−05	201.64
Mw (Gas)	gr/mole	24.8	28.05	7.97	2.44	16.04	78.11
Tb (Gas)	K	153.3	169.5	4.05	1.64	111.63	353.3
Pc (Gas)	bar	47.45	46	1.99	− 0.5	38	51.2
Tc (Gas)	K	255.7	283	3.83	1.57	190.55	563
ω (Gas)	–	0.06	0.086	0.065	0.75	0.011	0.212
Tc (IL)	K	1056.6	1189.9	− 0.56	0.188	643.1	1878.8
Pc (IL)	bar	21	21.59	0.2	− 0.2	5.13	40.46
Tb (IL)	K	772.11	839.7	0.4	0.57	495.2	1374.9
ω (IL)	–	0.59	0.585	0.111	0.072	− 0.2	1.43
Mw (IL)	gr/mole	392.9	419.4	0.887	0.623	135.16	928.88
Solubility	Mole fraction	0.069	0.0367	5.2	2.19	5E−08	0.496

**Table 3:** Different substructures of ionic liquids and hydrocarbons.

Substructures	
–CH_3_	–COO–
–CH_2_–	=O
>CH–	–NH_2_
>C<	–NH_3_
=CH_2_	> N–
=CH–	=N–
=C<	–F
=C=	–P
≡C–	–B
–OH	–S–
–O–	–SO_2_

## Modeling

### Multivariate adaptive regression spline (MARS)

The multivariate adaptive regression spline (MARS), which is comprised of a count of different basis functions, will be used for regressive assessment and the identification of estimation techniques. The MARS model can calculate the enhanced structure of the multiplication of spline basis functions (BFs). The MARS system may choose the count of BFs and the properties related to each one, like the multiplication degree and node positions, autonomously. We might mention recurrent regression as one of the various clustering techniques. To properly describe higher-order responses, the MARS model employs the backward stepwise approach^55,56^. The MARS concept has several strengths. These benefits provide several items that will be detailed further below. This model's practical property is that the count of parameters in this mathematical equation is reduced, which speeds up the calculation of the body while reducing simulation precision. Conserving computing resources is extremely desired for researchers. This model's clever method of dividing factors into two groups of crucial factors and supplementary factors, which maximize detection accuracy, is another noteworthy aspect. The mathematical look of this concept is one of its most appealing qualities^57^. The dependent and independent components are identified by the mathematical expression form^58^. There is no supposition for linking inputs and output among the premises utilized in the MARS system. The purpose of this analysis is to look into the relationship between the output y and inputs {x_1_,…, x_n_} based on previous revelations. The following is a description of the data generation system^59^:

1 $$y=f\left({x}_{1},\dots ,{x}_{n}\right)+e$$

*e* refers to the relative error that matches across the intended region (x_1_,…, x_n_) in which the MARS model is generated. A progressive linear regression technique is a frequent model-building approach that MARS employs. MARS employs an automated way to replace major inputs. A succession of comparable splines and their derivatives is created to provide these possibilities. The splines are coupled and form polynomial curves to construct an ideal model. BFs are the name for these polynomials. They can simulate both linear and nonlinear processes. BFs are often split into couples, each piecewise linear or cubic in its own right. They have nodes at a certain place, each of which is related to the inputs. Below equation gives the shape of distributed linear BFs as^59^:

2 $${\left(x-t\right)}_{+}=max\left(0,x-t\right)=\left\{\begin{array}{ll}x-t &\quad if \quad x>t\\ 0 &\quad otherwise\end{array}\right. \; \mathrm{and} \quad {\left(t-x\right)}_{+}=max\left(0,t-x\right)=\left\{\begin{array}{ll}t-x &\quad if \quad x<t\\ 0 &\quad otherwise\end{array}\right.$$

The positive component is denoted by “+”, while the node is denoted by “t”. The MARS model is calculated using the following formula in its standard form^59^:

3 $$f\left(X\right)={\beta }_{0}+\sum_{m=1}^{M}{\beta }_{m}{B}_{m}(X)$$

In the above equation, X is the input vector, and B_m_, β_m_, and β_0_ are mth BF, coefficient of mth BF, and the constant of the equation, respectively.

For the MARS approach, there are two stages to follow^59^:

**Stage 1:** In the targeted area, a collection of BFs is generated. This process begins with a structure that just has the intercept term and adds the phrases one at a time. BFs should be used for the phrases. The forward step can be stopped in two ways: the first is when the maximum number of BFs is reached, and the second is when the regression coefficient of modifications is less than the set value. Several BFs are consecutively utilized, resulting in an overfitting system.

**Stage 2:** Overfitting is undesired and must be avoided at all costs. Because the system uses a monitoring phrase, it must be condensed by deleting at least one more relevant BF at a time. Then, from among the best systems of any size, the system with the least general cross-validation (GCV) is chosen to complete the system. This procedure concludes with the creation of a final model. GCV is used in the MARS system to eliminate BFs that aren't needed. A GCV expression is as follows^60,61^:

4 $$GCV= \frac{{MSE}_{train}}{{\left(1-\frac{enp}{N}\right)}^{2}}$$

**Figure 2:**
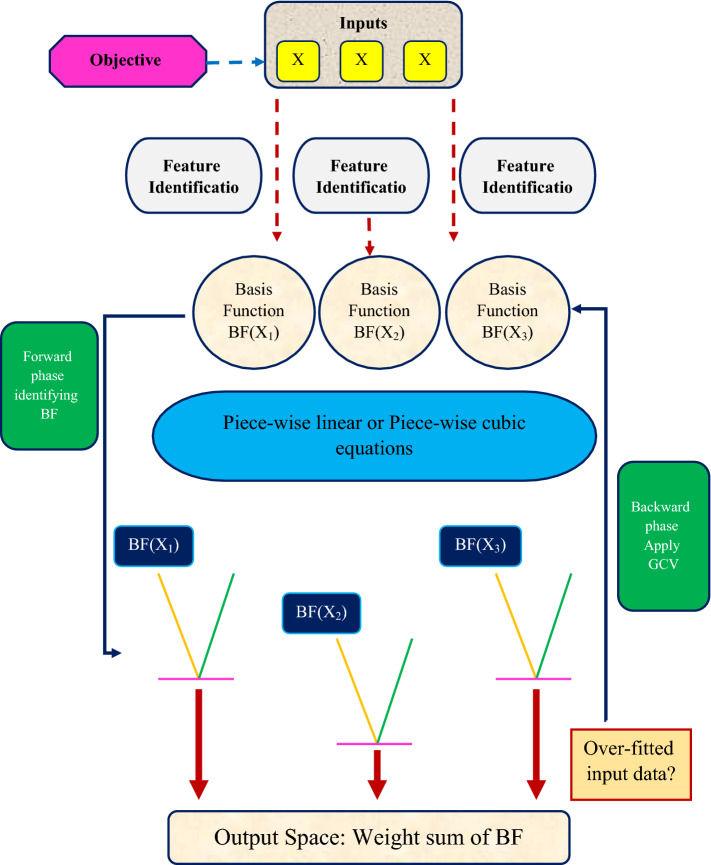
Schematic of the MARS model.

MSE_train_ represents the mean square error when utilizing training datasets, N represents the size of observations, and *enp* indicates the number of factors in the practical circumstance. Figure 2 illustrates the schematic of the MARS model.

### Extreme learning machine (ELM)

**Figure 3:**
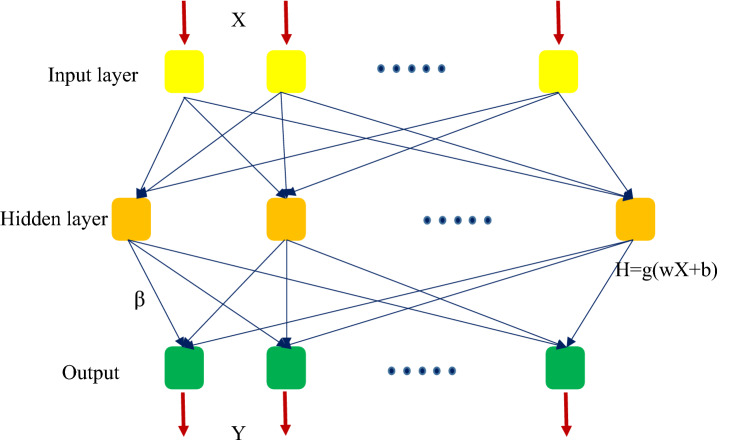
Schematic of the structure of ELM.

Huang G.B. invented the extreme learning machine (ELM) to minimize time-consuming repeated training and improve segmentation accuracy^62,63^. The ELM architecture consists of an input layer, a concealed layer, and an output layer. It is a single-hidden-layer feedforward neural network (SLFN). Unlike conventional learning methods (like the Backpropagation), which arbitrarily established all system training variables and quickly build local best solutions, the ELM just adjusts the neurons in the invisible layer, randomizes the weights between the visible and invisible layers, and also the bias of the hidden neurons in the method implementation stage, determines the hidden layer output matrix, and subsequently finds the weights. The ELM provides the prospect of quick learning speed because of its straightforward network architecture and succinct variable calculation operations. Figure 3 depicts the architectural framework of ELM. Figure 3 shows the construction of an extreme learning machine system, which has n input layer neurons, l hidden layer neurons, and m output layer neurons. Given the training set $\left\{X,Y\right\}=\left\{{x}_{i},{y}_{i}\right\} (i=1, \dots , Q)$, with an input parameter $X=\left[{x}_{i1} {x}_{i2} \dots {x}_{iQ}\right]$ and an output matrix $Y=\left[{y}_{i1} {y}_{i2} \dots {y}_{iQ}\right]$ composed of the training set, the matrix X and the matrix Y may be represented as follows^64^:

5 $$X=\left[\begin{array}{ccc}{x}_{11}& \cdots & {x}_{1Q}\\ \vdots & \ddots & \vdots \\ {x}_{n1}& \cdots & {x}_{nQ}\end{array}\right] ,Y=\left[\begin{array}{ccc}{y}_{11}& \cdots & {y}_{1Q}\\ \vdots & \ddots & \vdots \\ {y}_{m1}& \cdots & {y}_{mQ}\end{array}\right]$$

here, n and m are the dimensions of the input and output matrices, respectively.

The ELM then adjusts the weights between the input and hidden layers at random^64^:

6 $$w=\left[\begin{array}{ccc}{w}_{11}& \cdots & {w}_{1n}\\ \vdots & \ddots & \vdots \\ {w}_{l1}& \cdots & {w}_{ln}\end{array}\right]$$

The weights between the jth input layer neuron and the ith hidden layer neuron are represented by *w*_ij_.

Third, the ELM makes the following assumptions about the weights between the hidden and output layers^64^:

7 $$\beta =\left[\begin{array}{ccc}{\beta }_{11}& \cdots & {\beta }_{1m}\\ \vdots & \ddots & \vdots \\ {\beta }_{l1}& \cdots & {\beta }_{lm}\end{array}\right]$$

The weights between the jth hidden layer neuron and the kth output layer neuron are represented by ${\beta }_{jk}$.

Fourth, the ELM sets the buried layer neurons' bias at random^64^:

8 $$B={\left[{b}_{1} {b}_{2} \dots {b}_{n}\right]}^{T}$$

Fifth, the ELM selects the g(x) system activation function.

The output matrix T may be represented as follows according to Fig. 3:

9 $$T={\left[{t}_{1},{t}_{2}, \dots ,{t}_{Q}\right]}_{m\times Q}$$

The output matrix T's column vectors are as follows^64^:

10 $${t}_{j}=\left[\begin{array}{c}{t}_{1j}\\ \vdots \\ {t}_{mj}\end{array}\right]=\left[\begin{array}{c}\sum_{i=1}^{l}{\beta }_{i1}g\left({w}_{i}{x}_{j}+{b}_{i}\right)\\ \vdots \\ \sum_{i=1}^{l}{\beta }_{im}g\left({w}_{i}{x}_{j}+{b}_{i}\right)\end{array}\right],\quad \mathrm{ j}\hspace{0.17em}=\hspace{0.17em}1,\dots ,\mathrm{ Q}$$

Sixth, have a look at Eqs. (9) and (10) to see what we can come up with:

11 $$H\beta ={T}^{^{\prime}}$$

here, ${T}^{^{\prime}}$ is the transpose of T and H is the concealed layer's output. We utilize the least square approach to determine the weight matrix values of $\beta$^62^ to find a particular solution with the least amount of error.

12 $$\beta ={H}^{+}{T}^{^{\prime}}$$

We apply a regularization element to the $\beta$^65^ to increase the network's generalization capabilities and make the findings more robust. When the count of concealed layer neurons is smaller than the number of training sets, $\beta$ can be calculated as:

13 $$\beta ={{\left(\frac{I}{\lambda }+{H}^{T}H\right)}^{-1}}H^{T}{T}^{^{\prime}}$$

When the number of concealed layer nodes exceeds the number of training data, $\beta$ is^64^:

14 $$\beta ={H}^{T}{{\left(\frac{I}{\lambda }+{H}^{T}H\right)}^{-1}}^{T}{T}^{^{\prime}}$$

### Support vector regression (SVR)

Smola and Scholkopf^66^ have written a detailed description of SVR methodology. As a result, this study mainly provides a basic overview of SVR. SVR fits the linear relationship $y={x}^{T}p+b$ regarding the designation error reduction theory, in which the regression parameters *p* and *b* are determined by minimizing the given cost function *R*^67^:

15 $$R= \frac{1}{2}{P}^{T}P+C\frac{1}{J}\sum_{j=1}^{J}{L}_{\varepsilon }\left({d}_{j},{y}_{j}\right)$$

16 $${L}_{\varepsilon }\left({d}_{j},{y}_{j}\right)=\left\{\begin{array}{ll}\left|{d}_{j},{y}_{j}\right|-\varepsilon &\quad \left|{d}_{j},{y}_{j}\right|\ge \varepsilon \\ 0 &\quad otherwise\end{array}\right.$$

here, d_j_ and y_j_ refer to the measured and predicted values of the jth training component, respectively, and J is the count of training components. This cost function aims to minimize both model intricacy (1/2(P^T^P)) and actual error ($C\frac{1}{J}\sum_{j=1}^{J}{L}_{\varepsilon }\left({d}_{j},{y}_{j}\right)$) at the same time. C is a regularization coefficient that controls the model intricacy vs. actual error trade-off. ${L}_{\varepsilon }\left({d}_{j},{y}_{j}\right)$ is the $\varepsilon$-insensitive loss function, as stated in Eq. (16). The cost function *R* is limited in the following scenario when slack parameters ξ are introduced^67^:

17 $$R= \frac{1}{2}{P}^{T}P+C\sum_{j=1}^{J}\left({\upxi }_{j},{\upxi }_{j}^{*}\right), \to \left\{\begin{array}{l}{d}_{j}-{x}_{j}^{T}P-b\le \varepsilon +{\upxi }_{j}\\ {-d}_{j}+{x}_{j}^{T}P+b\le \varepsilon +{\upxi }_{j}^{*}\\ {\upxi }_{j}^{*},{\upxi }_{j}\ge 0\end{array}\right.$$

Therefore, by employing the optimization criteria and inserting Lagrange multipliers $\left({\alpha }_{j}^{*},{\alpha }_{j}\right)$ linear relationship $y={x}^{T}p+b$ has the given explicit expression^67^:

18 $$y= \sum_{j=1}^{J}\left({\alpha }_{j}-{\alpha }_{j}^{*}\right)\langle {x}_{j},x\rangle +b$$

**Figure 4:**
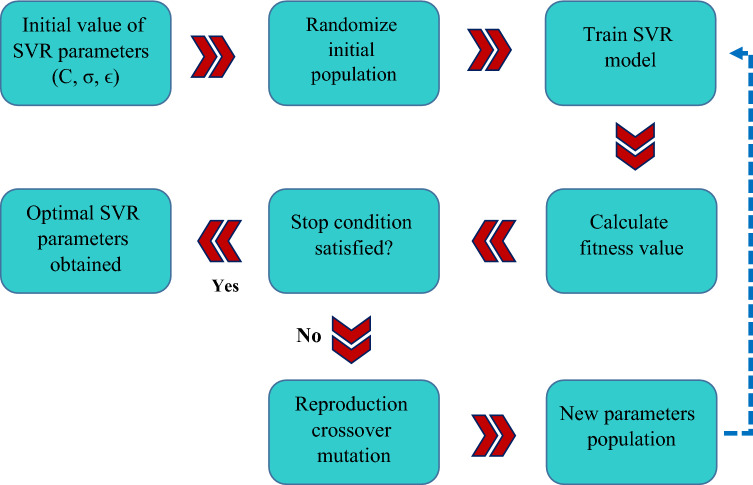
Flowchart of the SVR model.

By replacing a kernel function $\mathrm{K}\left({x}_{j},\mathrm{x}\right)$ for the dot product $\langle {x}_{j},x\rangle$, this linear approach may be extended to nonlinear regression in a direct way. As the kernel function, a variety of functions can be employed. The Gaussian radial basis function $\mathrm{K}\left({x}_{j},\mathrm{x}\right)=\mathrm{exp}\left(-\Vert {x}_{j}-x\Vert /\left(2{\gamma }^{2}\right)\right)$ is a regularly utilized kernel function^67^. Figure 4 shows the flowchart of the SVR approach.

### Boosting support vector regression (B-SVR)

For the regression problem, many implementations of boosting were utilized, including adaptive boosting for regression^68^ and Stochastic gradient boosting^69^. The essential objective of B-SVR is to train a series of SVR systems, each of which is built often using data with high mistakes gained from the prior SVR designs. The following is a description of the technique^67^.

To begin, all of the data in the initial training dataset are given identical weights ${w}_{j}^{(1)}=1, j=1,\dots ,J$.

Then, for each computation phase t = 1,…,T (where T is the group size or repetition number), do the foregoing:

(First Stage): The probability of drawing the ith input for SVR analysis is^67^:

19 $${f}_{j}^{(t)}=\frac{{w}_{j}^{(t)}}{\sum_{j=1}^{J}{w}_{j}^{(t)}}, \quad j=1,\dots ,J.$$

*J* samples (sometimes with repetition, i.e., some values that occur several times in the boosting collection) are chosen from the main training samples according to the selection probability functions *f*(*t*).

(Second stage): Utilizing boosting dataset, create an SVR network and predict the outputs of the main training data.

(Third stage): Using the given loss function, calculate a loss value for each item in the initial training dataset^67^:

20 $${L}_{j}=\frac{\left|{y}_{j}^{(t)}-{d}_{j}\right|}{\mathrm{max}({y}^{(t)}-d)}, \quad j=1,\dots ,J.$$

(Fourth stage): Determine the mean loss:

21 $${\overline{L} }_{t}=\sum_{j=1}^{J}{L}_{j}{f}_{j}$$

(Fifth stage): Consider ${\beta }_{t}=\frac{{\overline{L} }_{t}}{1-{\overline{L} }_{t}}$ and modify the weight of each component in the previous training dataset utilizing^67^:

22 $${w}_{j}^{(t+1)}={w}_{j}^{(t)}{\beta }_{t}^{(1-{L}_{j})}$$

The notation (1 *− L*_*j*_) indicates that ${\beta }_{t}$ has been increased to the (1 *− L*_*j*_)th power. This weight-updating technique indicates that if the SVR system generated in the second stage fails to estimate data, its weight will be raised. ${\beta }_{t}$ is a measure of the developed SVR model's reliability. A high ${\beta }_{t}$ suggests that the SVR system has a poor level of certainty. Because the quantity of ${w}_{j}^{(t+1)}$ is required to calculate ${f}_{j}^{(t+1)}$ in the first stage of the next computation phase (*t* + *1*), Eq. (22) yields a transition to the next calculation cycle (t + 1). T is chosen by the training collection's root mean squared error (RMSE)^70^, and boosting cycle is performed by Drucker^71^ when the average loss is less than 0.5^67^.

Finally, in terms of estimation, each of the T SVR systems produces a forecasting ${y}_{j}^{(t)}$ for the jth unknown feature and a matching ${\beta }_{t}$. These T forecasts are merged to provide a target variable utilizing the weighted median method, which is calculated as follows:

Arrange these T forecasts, ${y}_{j}^{(t)}$ (t = 1,…, T), in increasing sequence for the jth sample ^67^:

23 $${y}_{j}^{(n1)}\le {y}_{j}^{\left(n2\right)}\le \dots \le {y}_{j}^{\left(nJ\right)}$$

here, n_j_ (j = 1, 2,…, J; J = T) is the initial cycle number t for the jth sample's associated forecast. Consider the case where the biggest predicted value of y_i_ was anticipated by the third computation cycle (t = 3), in which case ${y}_{j}^{(nJ)}$ should equal ${y}_{j}^{(3)}$. From the lowest term n_1_ to the rth term n_r_, add the sum of $\mathrm{log}(\frac{1}{{\beta }_{nj}})$ through *j* until the following inequality is satisfied^67^:

24 $$\sum_{j=1}^{r}\mathrm{log}(\frac{1}{{\beta }_{nj}})\ge \frac{1}{2}\sum_{j=1}^{J}\mathrm{log}\left(\frac{1}{{\beta }_{nj}}\right)$$

The overall estimate for the jth data is then extracted from the forecast from the n_r_th SVR system^71^. Utilizing the same technique as before, group estimates of outputs for additional inputs may also be produced^67^.

### Deep belief network (DBN)

Artificial Neural Networks (ANNs) have been initially suggested in the 1960s and now have grown in prominence in a variety of machine learning approaches, including classification, clustering, regression, and forecasting^72^. ANNs are flexible analytical approaches that can build complicated associations between real-world information. Among the several types of ANNs, the Back-Propagation (BP) mechanism is one of the most common structures. Although, it should be highlighted that using casual weights at the beginning of the training process is one of the difficulties with BP. As a consequence of this challenge, a unique technique, Deep Belief Network (DBN), for deep network pre-training was devised in 2006^73^, leading to significant advances in machine learning.

**Figure 5:**
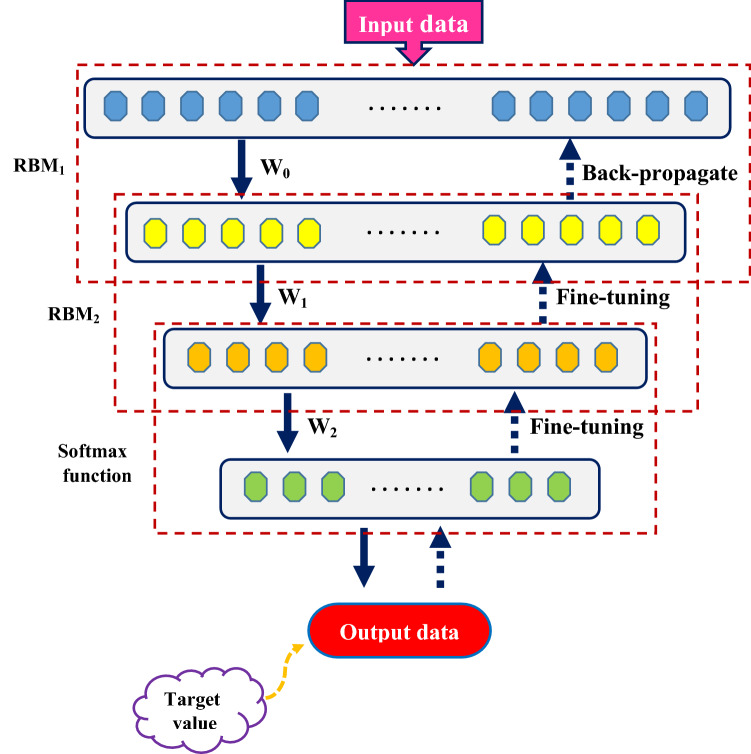
The DBN algorithm flowchart.

A DBN is comprised of several Restricted Boltzmann Machines (RBMs), which are unattended creative prediction model that uses one hidden layer to mimic the presumed distribution of measurable variables. Our DBN uses a series of RBMs to identify relevant architectures, which extract high-level properties from original data. Figure 5 shows a DBN in graphical form^74^.

DBN training is divided into three phases^75^:

1. Conduct unsupervised and hungry layer-wise pre-training utilizing a collection of RBMs.
2. After haphazardly setting the linking weights matrix between the last hidden layer and the output neuron, calculate the error (first fine-tuning stage).
3. As a second fine-tuning stage, use error Back Propagation. The RBM training technique is described as follows:

#### Restricted Boltzmann machine

**Figure 6:**
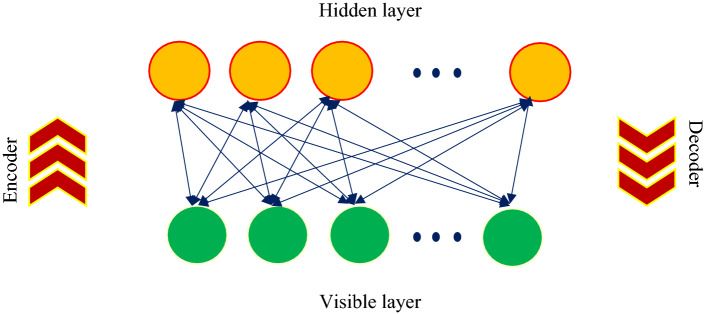
Schematic of the restricted Boltzmann machine.

The encoder/decoder technique used by the Restricted Boltzmann Machine turns inputs into an increased attributes statement (Fig. 6). The decoder may then retrieve the inputs. RBM growth through the reproduction of inputs is a major feature of DBN because this strategy is unregulated and does not need marked data^76^.

The RBM is made up of a number of visible, v ∈ {0,1}^gv^, and concealed units, h ∈ {0,1}^gh^, the number of which is represented by the gv and gh, respectively. The energy of the joint link {v,h} in RBM, considering bias, is^77^:

25 $$E\left(v.h\right)=-{v}^{T}Wh-{b}^{T}v-{c}^{T}h$$

The vectors of visible units, hidden units, visible unit's bias, and hidden unit's bias are denoted by x, h, b, and c, respectively. The transpose vectors of v, b, and c are denoted by v^T^, b^T^, and c^T^, respectively. The weight matrix is also W. The following formula is used to compute the probability of each possible situation {v,h}v^76^:

26 $$P\left(v.h\right)=\frac{1}{Z}\mathrm{exp}\left(-E\left(v.h\right)\right)$$

here, Z is the normalizing factor and denotes:

27 $$Z=\sum_{v.h}exp\left(-E(v.h)\right)$$

In this work, we have used intelligent models based on two different methods. The reason for choosing the thermodynamic properties-based method is to make a model whose inputs are the same as the equations of state so that it is simple and a reasonable comparison can be made. The reason for choosing the method based on chemical structure is to make a model that is more general and can be used for different ionic liquids and operating conditions.

## Equations of state (EOSs)

Equations of state (EOSs) are utilized to determine the thermodynamic characteristics of pure substances and mixtures, particularly for processes at pressures higher than 10 bar, since in these circumstances at least one constituent exists in supercritical phase in most cases. The cubic EOSs are the most common types of EOS. The van der Waals EOS, proposed in 1873^78^ as the first model to represent features of both liquid and vapor phases, is the source of these approaches. Numerous EOS have been built on top of the van der Waals EOS in recent decades. For instance, there are over 220 versions and numerous researches that are relevant to parameterization and application to compounds for the widely used Peng–Robinson EOS. Cubic EOS has become quite widespread in the oil and gas industry because they are straightforwardly formulated mathematically and provide good thermodynamic property relationships and forecasts for compounds of different fluids^79^. In this study, to evaluate the performance of intelligent techniques, the obtained results are compared with equations of state. In this regard, four equations of state including Peng–Robinson (PR), Soave–Redlich–Kwong (SRK), Redlich–Kwong (RK), and Zudkevitch–Joffe (ZJ) have been used. The mathematical relations of the equations mentioned are summarized in Table 4.

**Table 4:** Equations of state applied in this work.

EOS	Reference	Relationship	Coefficients
PR3: 3-parameter Peng–Robinson	^88^	$P=\frac{RT}{v-b}-\frac{a}{{v}^{2}+uv+w}$	$a={\Omega }_{a}\frac{{R}^{2}{T}_{C}^{2}}{{P}_{c}}$
			${\Omega }_{a}$=${\Omega }_{ac}\alpha \left({T}_{r}\right)$
			$$b={\Omega }_{b}\frac{R{T}_{c}}{{P}_{c}}$$
			$$C={\Omega }_{c}\frac{R{T}_{c}}{{P}_{c}}$$
			$$u={\Omega }_{u}\frac{R{T}_{c}}{{P}_{c}}$$
			$$w={\Omega }_{w}{\left(\frac{R{T}_{c}}{{P}_{c}}\right)}^{2}$$
			$$\alpha ={\left[1+m(1-{{T}_{r}}^{0.5})\right]}^{2}$$
SRK3: 3-parameter Soave–Redlich–Kwong	^89^	$P=\frac{RT}{v-b}-\frac{\alpha a}{v\left(v+b\right)}$	$a=\frac{0.42747{R}^{2}{T}_{c}^{2}}{{P}_{c}}$
			$$b=\frac{0.08664R{T}_{c}}{{P}_{c}}$$
			$$\alpha ={\left[1+m(1-{{T}_{r}}^{0.5})\right]}^{2}$$
			$$m=0.48508+1.5517\omega -0.1561{\omega }^{2}$$
RK: Redlich–Kwong	^90^	$P=\frac{RT}{v-b}-\frac{ a}{\sqrt{T}v\left(v+b\right)}$	$a=\frac{0.42748{R}^{2}{T}_{c}^{2.5}}{{P}_{c}}$
			$$b=\frac{0.08664R{T}_{c}}{{P}_{c}}$$
ZJ: Zudkevitch–Joffe	^91^	$P=\frac{RT}{v-b}-\frac{ a}{\sqrt{T}v\left(v+b\right)}$	Parameter *a* and *b* are calculated as a functions of temperature and pressure

## Results and discussion

### Statistical analysis

**Figure 7:**
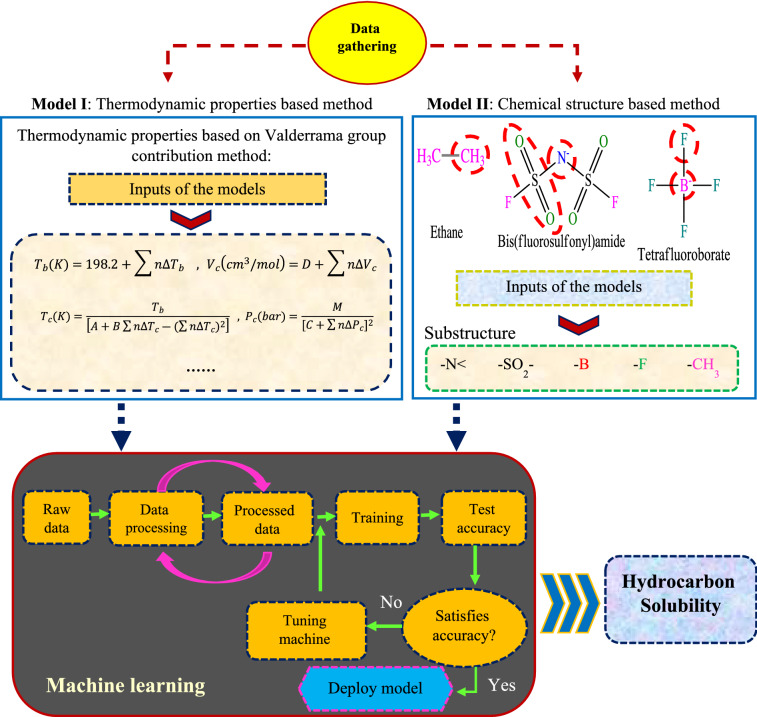
Procedure of data preparation and model development for hydrocarbon solubility prediction.

In this study, the solubility of gaseous hydrocarbons in ILs was predicted using 2145 laboratory data with the four intelligent models already mentioned. For this purpose, two different methods based on thermodynamic properties and the chemical structure of ILs and hydrocarbons have been used. The process of model development and estimation of the solubility values is illustrated in Fig. 7. Statistical criteria comparing the performance of intelligent models are root mean squared error (RMSE) and coefficient of determination (R^2^), the mathematical relationships of which are defined as follows^24,80^:

28 $$RMSE=\sqrt{\frac{1}{n}\sum_{i=1}^{n}{\left({HS}_{i exp}-{HS}_{i pred}\right)}^{2}}$$

29 $${R}^{2}=1-\frac{\sum_{i=1}^{n}{\left({HS}_{i exp}-{HS}_{i pred}\right)}^{2}}{\sum_{i=1}^{n}{\left({HS}_{i exp}-{\overline{HS} }_{i exp}\right)}^{2}}$$

where ${HS}_{i exp}$, ${HS}_{i pred}$, ${\overline{HS} }_{i exp}$, and n refer to experimental hydrocarbon solubility, predicted hydrocarbon solubility, mean value of experimental hydrocarbon solubility, and total number of data points, respectively.

The results of statistical analysis of the developed models are reported in Table 5. According to the obtained results, the DBN model has the best performance in both methods. In the method based on thermodynamic properties, the DBN model has the highest accuracy with R^2^ and RMSE values of 0.9961 and 0.0054, respectively. The R^2^ and RMSE values for the DBN model in the chemical structure-based method are 0.9943 and 0.0065, respectively. Generally, the developed models are arranged in the following order of increasing accuracy:

**Table 5:** Statistical analysis results of developed models.

Model	Data	Statistical parameter			
		Model (I)		Model (II)	
		RMSE	R^2^	RMSE	R^2^
Boosting-SVR	Train	0.0083	0.9906	0.0075	0.9923
	Test	0.0119	0.9825	0.0110	0.9849
	Total	0.0091	0.9889	0.0083	0.9907
DBN	Train	0.0033	0.9985	0.0055	0.9959
	Test	0.0101	0.9874	0.0097	0.9883
	Total	0.0054	0.9961	0.0065	0.9943
ELM	Train	0.0052	0.9963	0.0052	0.9964
	Test	0.0105	0.9863	0.0111	0.9846
	Total	0.0066	0.9942	0.0068	0.9938
MARS	Train	0.0135	0.9752	0.0104	0.9851
	Test	0.0169	0.9646	0.0152	0.9714
	Total	0.0142	0.9729	0.0115	0.9822

- Model (I): MARS < B-SVR < ELM < DBN.
- Model (II): MARS < B-SVR < ELM < DBN.

### Graphical analysis

**Figure 8:**
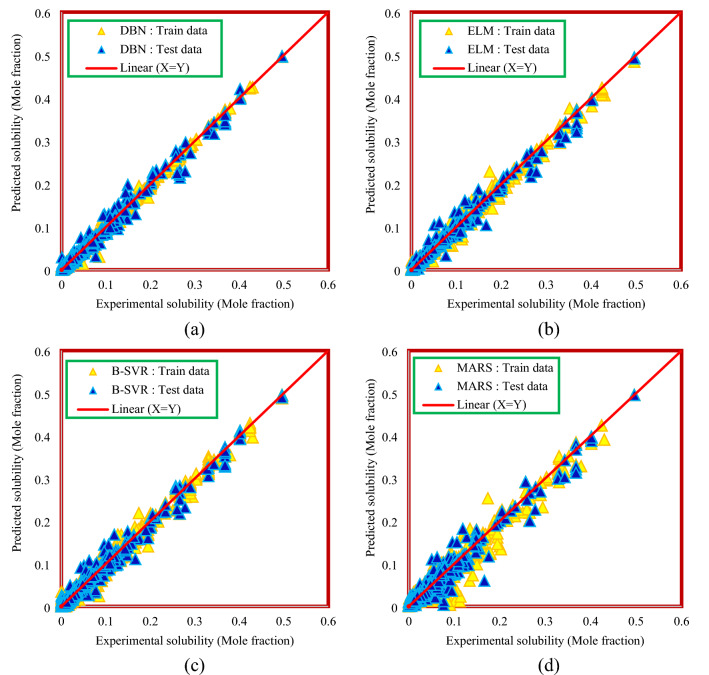
Cross plot diagram of the results of Model (I): (**a**) DBN model, (**b**) ELM model, (**c**) B-SVR model, (**d**) MARS model.

**Figure 9:**
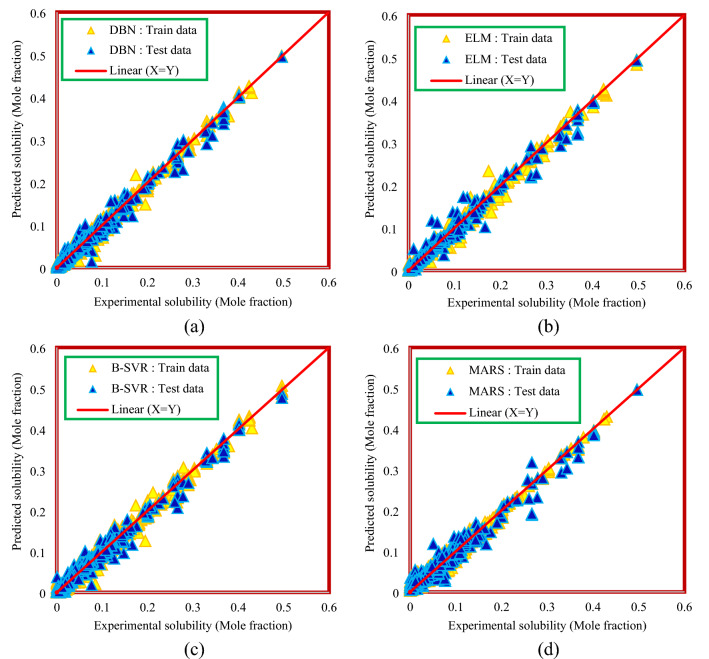
Cross plot diagram of the results of Model (II): (**a**) DBN model, (**b**) ELM model, (**c**) B-SVR model, (**d**) MARS model.

Plotting the predicted values versus the experimental values obtain a cross plot diagram. In this diagram, the more points that correspond to the X = Y line, the closer the predicted values are to the actual values, so the accuracy of the model will increase. Figure 8 shows a cross-plot diagram for models developed based on thermodynamic properties. As can be seen from Fig. 8, the DBN model has higher density points around the X = Y line, which indicates the higher accuracy of this model than other models, although other models are also in an acceptable situation. Figure 9 shows the cross-plot diagram of the results of the method based on chemical structure, temperature, and pressure. The graphical analysis presented in Fig. 9 also confirms the results of Table 5, and the DBN model has a more appropriate cross plot diagram than the other models.

**Figure 10:**
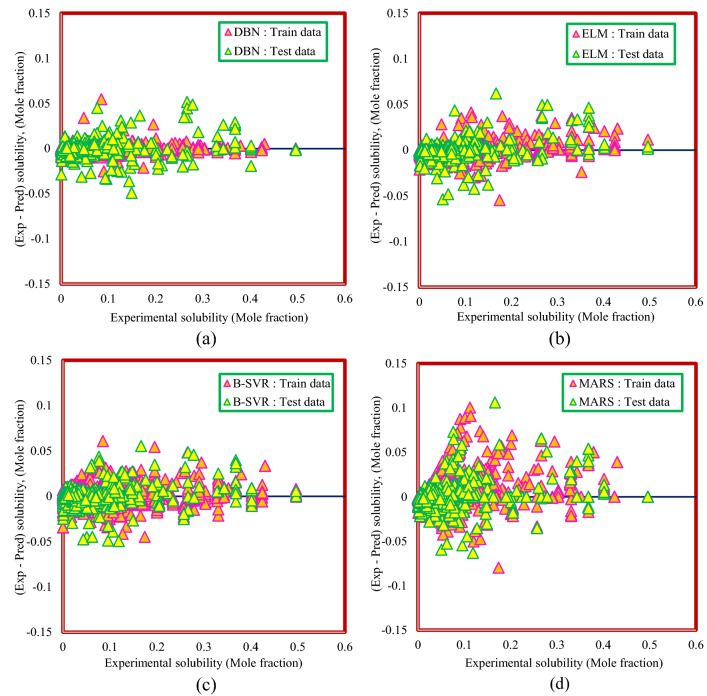
Distribution of deviation of predicted values from actual values for Model (I): (**a**) DBN model, (**b**) ELM model, (**c**) B-SVR model, (**d**) MARS model.

**Figure 11:**
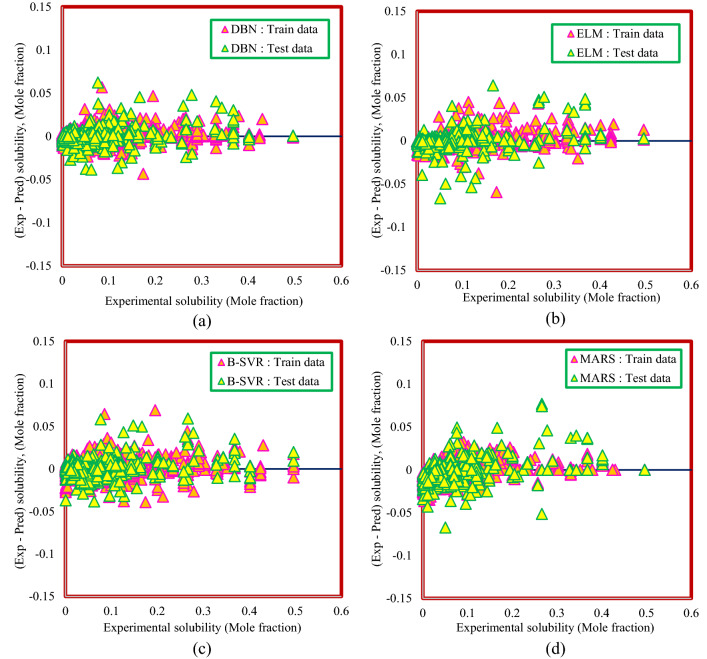
Distribution of deviation of predicted values from actual values for Model (II): (**a**) DBN model, (**b**) ELM model, (**c**) B-SVR model, (**d**) MARS model.

Figures 10 and 11 illustrate the distribution of the deviation of the predicted values from the actual values for Model (I) and Model (II), respectively. The higher the focus around the zero line, the closer the predicted values are to the actual values and the more accurate the model. Therefore, as can be seen from Figs. 10 and 11, the DBN model is in a better position than the other smart models in both methods.

**Figure 12:**
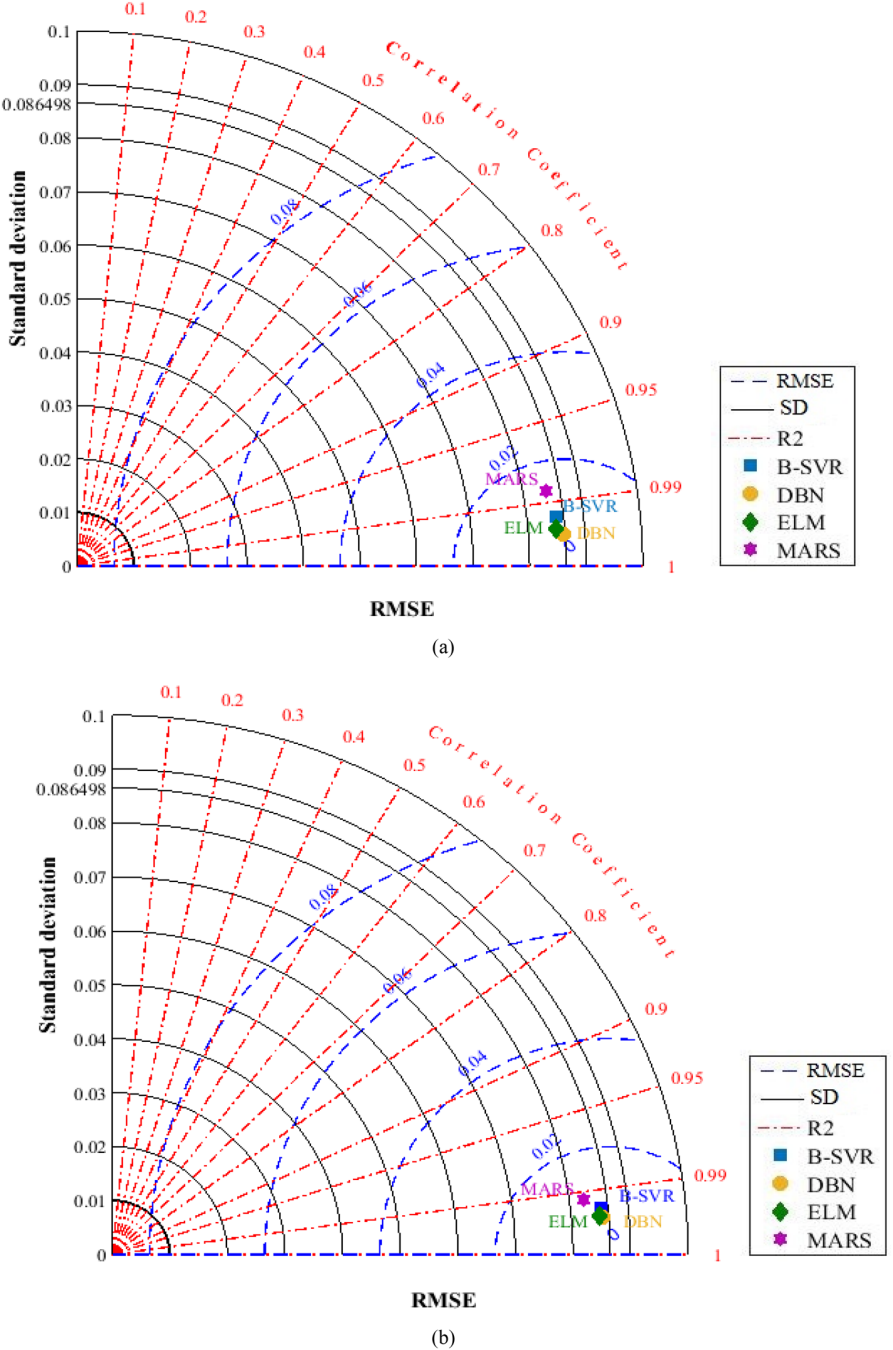
Taylor diagram of developed models: (**a**) Model (I), (**b**) Model (II).

The Taylor diagram^81^ was developed in Fig. 12, which integrates a number of statistical variables for a more understandable representation. This illustration depicts all of the smart approaches in respect of how well they forecast gaseous hydrocarbon solubility in various ILs. Three performance parameters [standard deviation (SD), RMSE, and R^2^] of each network, including DBN, ELM, B-SVR, and MARS, are utilized to quantify the degree of discrepancy between the systems' estimates and the related actual values. The reference point is a distance from the full measure that is used to calculate the centered RMSE. Another reference is the optimal estimation technique, which is denoted by a point with R^2^ equal to 1. Figure 12a,b show the Taylor diagram of the DBN model for the results of Model (I) and Model (II), respectively. In Fig. 12a, the RMSE and R^2^ values are 0.0054 and 0.9961, respectively. In Fig. 12b, the RMSE and R^2^ values are 0.0065 and 0.9943, respectively. Figure 12 depicts the performance parameters of the systems after they have been encapsulated within the Taylor diagram. The Taylor diagram in this picture verifies DBN's dominance by demonstrating that their worldwide performance assessment is the most accurate.

### Equations of state evaluation

**Figure 13:**
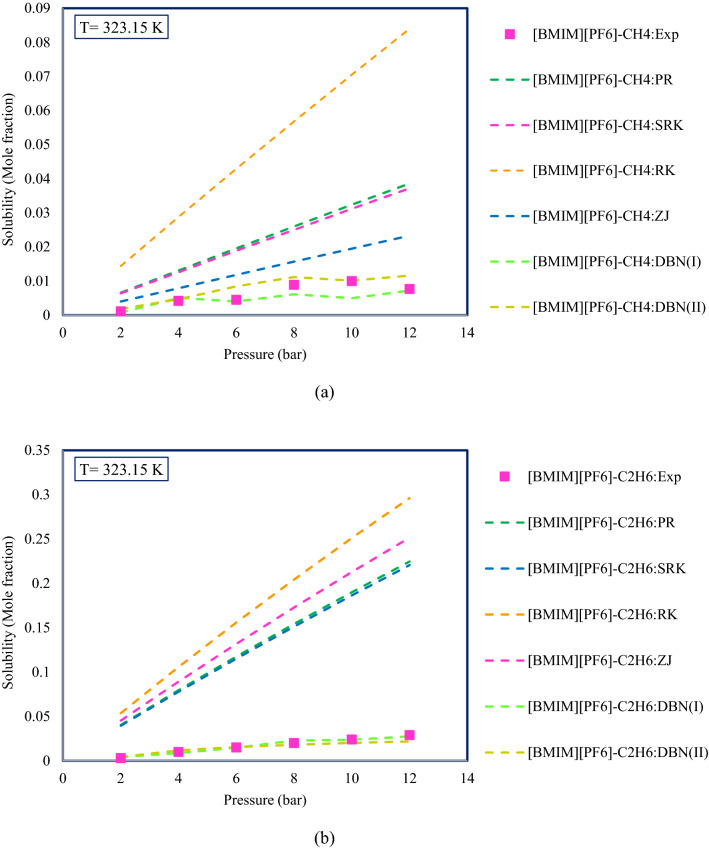
Comparison of the results of the DBN models and equations of state; (**a**) [BMIM][PF_6_]-CH_4_, (**b**) [BMIM][PF_6_]-C_2_H_6_.

Equations of state are one of the conventional methods for calculating the solubility of gases in ILs. Comparing the results of smart models with equations of state can reveal the efficiency and performance of smart models well. For this purpose, four equations of state including Peng–Robinson (PR), Soave–Redlich–Kwong (SRK), Redlich–Kwong (RK), and Zudkevitch–Joffe (ZJ) have been used to calculate the solubility of gaseous hydrocarbons in ILs. Figure 13 shows the results obtained for two systems ([BMIM][PF_6_]-CH_4_ and [BMIM][PF_6_]-C_2_H_6_) at 323.15 K. As can be seen from Fig. 13, with increasing pressure, the solubility of gaseous hydrocarbons increases, a trend that can also be seen in the equations of state. Also, the solubility of C_2_H_6_ in [BMIIM][PF_6_] is higher compared to CH_4_. Another noteworthy point is the very good agreement of the results of the smart models with the experimental data. It can also be found that the equations of state overestimate the solubility and the difference between the calculated values and the experimental data is also significant.

**Figure 14:**
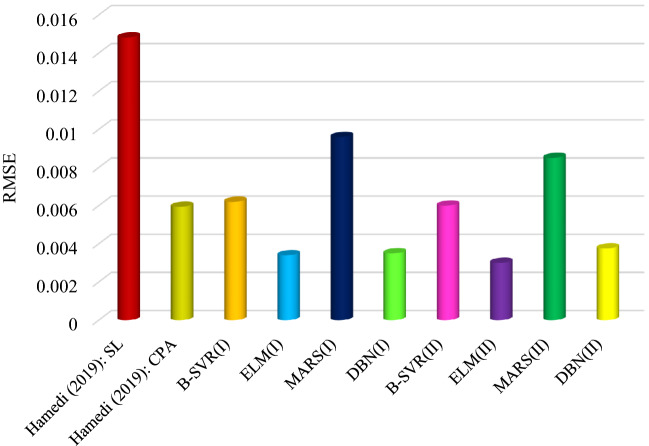
Comparison of the results of the current study models and equations of state used in previous studies.

Hamedi et al.^5^ calculated the solubility of methane in ILs using two equations of state including modified Sanchez and Lacombe (SL) and cubic-plus-association (CPA). In this study, 649 experimental data related to 19 ionic liquid and methane systems were used. Figure 14 shows a comparison of current study models and Hamedi et al. results. As can be seen, the accuracy of the intelligent models compared to the equations of state used by Hamedi et al.^5^ is significant. The advantages of the proposed models in the current study over the work of Hamedi et al. are the use of a wider database (2145 experimental data) including more diverse systems (48 systems) and more advanced general models. Also, the results obtained in this study are more accurate than the research of Hamedi et al.

### Trend analysis

Involvement of various parameters such as temperature, pressure, intermolecular interactions, etc. in the solubility of gases in ILs makes it difficult to find physical phenomena affecting the transfer process, but in simpler terms, changing solubility factors (such as Henry's constant) due to changes in physical and chemical properties cause these trends^82,83^. Enthalpy, entropy, and interactions between gas and ionic liquid are the most important factors affecting the solubility of gases in ILs. Therefore, the effect of changes in pressure, temperature, type of anion and cation on solubility is affected by changes in enthalpy and entropy. In other words, the endothermic or exothermic nature of the dissolution process, which is also depends on the interactions between the gas and the ionic liquid, affects how the gas solubility changes with the increase of each of the mentioned parameters^84^.

#### Pressure and anion type effect

**Figure 15:**
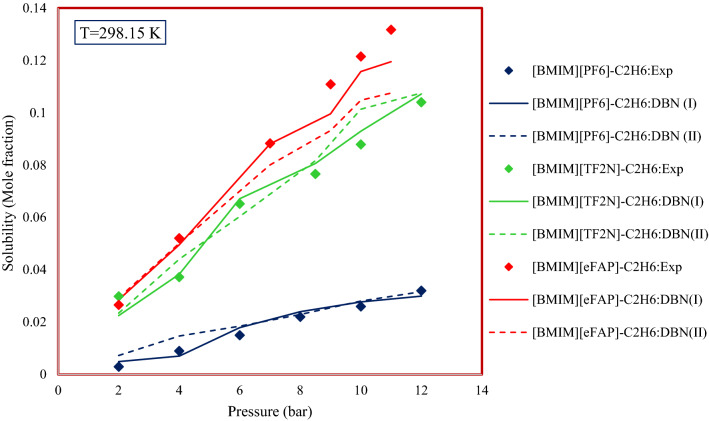
Pressure and anion type effect on the solubility of gaseous hydrocarbons in ionic liquids.

Previous studies have shown that although ILs have similar solubility at low pressures^85^, increasing pressure leads to increasing the solubility of gases in ILs and the rate of increase varies for different ILs^86^. Figure 15 shows the changes in the solubility of gaseous hydrocarbons with increasing pressure for a number of systems used in this study. As can be seen, increasing the pressure increases the solubility of gaseous hydrocarbons in ILs. The effect of the type of anions existing in the structure of ILs has been studied in many studies^86^. As mentioned in the literature, the main reasons for changes in the solubility due to changes in pressure and anion type are enthalpy and entropy changes, and the interactions between gas and ILs^84^. The effect of a number of anions has been investigated for example. Investigation of the systems reported in Fig. 15 shows that the effect of anion type on the solubility of C_2_H_6_ in ILs is as follows:

$$\left[ {{\text{BMIM}}} \right]\left[ {{\text{PF}}_{{6}} } \right] \, < \, \left[ {{\text{BMIM}}} \right]\left[ {{\text{TF}}_{{2}} {\text{N}}} \right] \, < \, \left[ {{\text{BMIM}}} \right]\left[ {{\text{eFAP}}} \right].$$

#### Cation effect

**Figure 16:**
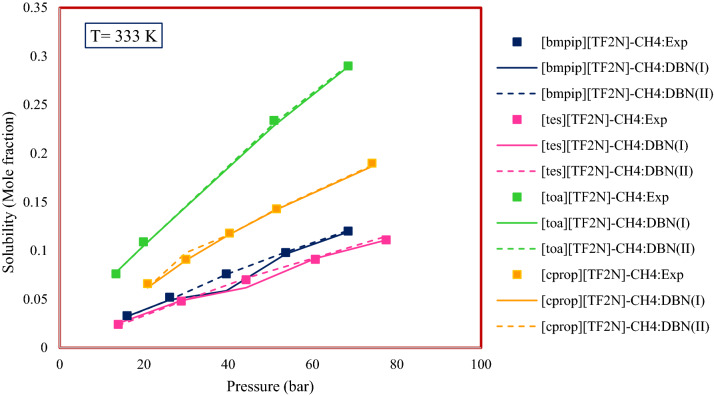
Cation type effect on the solubility of gaseous hydrocarbons in ionic liquids.

In previous studies, the influence of cation type has been investigated^43^. The reasons for the effect of cation type on solubility are similar to the reasons mentioned for the effect of anion type. In this study, the effect of a number of cations on the solubility of gaseous hydrocarbons in ILs has been studied. Figure 16 examines a number of systems studied in this work. According to the results obtained for the proposed systems, the effect of cation type on the solubility of methane in ILs is as follows:

$$\left[ {{\text{tes}}} \right]\left[ {{\text{TF}}_{{2}} {\text{N}}} \right] \, < \, \left[ {{\text{bmpip}}} \right]\left[ {{\text{TF}}_{{2}} {\text{N}}} \right] \, < \, \left[ {{\text{cprop}}} \right]\left[ {{\text{TF}}_{{2}} {\text{N}}} \right] \, < \, \left[ {{\text{toa}}} \right]\left[ {{\text{TF}}_{{2}} {\text{N}}} \right].$$

#### Temperature and alkyl chain length effect

**Figure 17:**
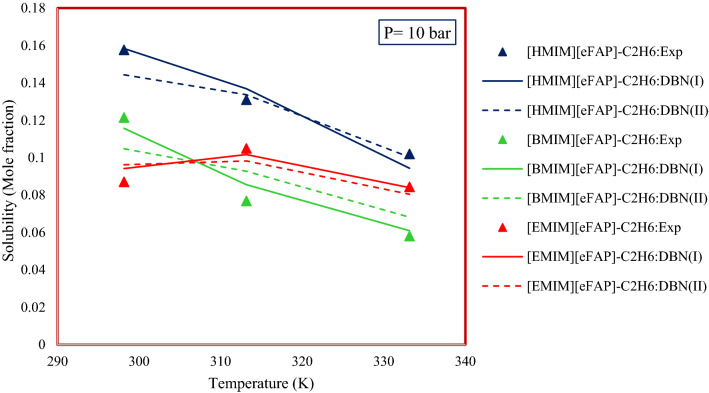
Temperature and alkyl chain length effect on the solubility of gaseous hydrocarbons in ionic liquids.

Many researchers have studied the effect of temperature^86^ and alkyl chain length^87^ on the solubility of gases in ILs. The results show that increasing the temperature reduces the solubility^86^. Generally, the dependency of solubility on temperature is thermodynamically related to the enthalpy of dissolution. For example, in exothermic processes (such as dissolution of CO_2_ in ILs) the solubility of the gas decreases with increasing temperature, and in endothermic processes (such as dissolution of N_2_ in ILs) the increase in temperature increases the solubility^83,84^. Also, increasing the alkyl chain length increases the solubility of hydrocarbons in ILs^87^. The reason for this subject is the increased entropy of solvation for ILs^84^. According to Fig. 17, although increasing the alkyl chain length does not show a specific trend in ethane solubility in the proposed systems, increasing the temperature has reduced the ethane solubility in the proposed systems.

#### Effect of the type of hydrocarbon

**Figure 18:**
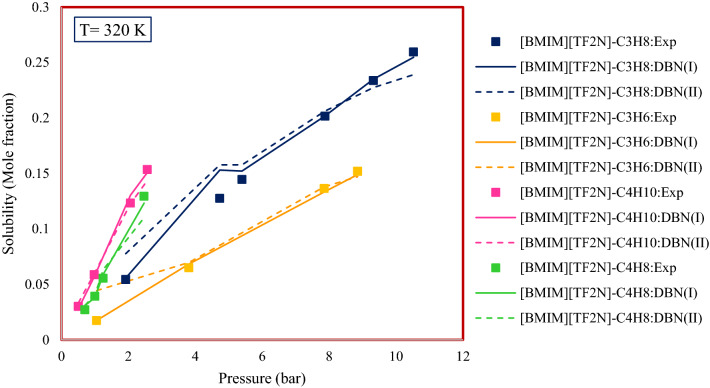
Type of hydrocarbon effect on the solubility of gaseous hydrocarbons n ionic liquids.

In order to investigate the effect of hydrocarbon type on the solubility of gaseous hydrocarbons, four different systems have been investigated. Figure 18 shows the trend of solubility changes for these four systems with increasing pressure at 320 K. According to the results, it can be seen that the solubility of proposed hydrocarbons in [BMIM][TF_2_N] is determined as follows:

$${\text{C}}_{{3}} {\text{H}}_{{6}} < {\text{ C}}_{{3}} {\text{H}}_{{8}} < {\text{ C}}_{{4}} {\text{H}}_{{8}} < {\text{ C}}_{{4}} {\text{H}}_{{{1}0}} .$$

Therefore, it can be concluded that increasing the molecular weight of hydrocarbons can increase their solubility in ILs.

Therefore, as it was investigated, different parameters can affect the solubility of gaseous hydrocarbons in ionic liquids, and this effect can vary depending on the type of gas and ionic liquid. For example, Ramdin et al.^4^ reported that although the enthalpy of absorption of CO_2_ is higher in [amim][dca] than in [thtdp[phos], its solubility is lower in [amim][dca]. On the other hand, this effect was the opposite for CH_4_, and the solubility of methane in ionic liquids corresponded to the enthalpy of adsorption. Also, its solubility in ionic liquids with longer non-polar alkyl chains is higher. Thus, entropic effects and solvent–solvent interactions prevailed for CO_2_ and CH_4_, respectively. The solubility of hydrocarbons in ILs is important in many ways. The selection of ILs as solvents in reactions involving permanent gas and gas storage media is strongly influenced by the degree of gas solubility in ILs and is essential for the development of separation methods. Investigation of the effect of various parameters such as temperature, pressure, etc. is necessary to deal with the separation of CO_2_ from natural gas as well as the low adsorption of light hydrocarbons in ILs such as methane, ethane and propane. Therefore, evaluating the effect of temperature, pressure, anion and cation type, hydrocarbon type, and alkyl chain length can help researchers to select the appropriate ILs in different operating conditions. The findings of the study also show that ILs are suitable candidates for the solubility of hydrocarbons so they can be used in various operational applications.

## Conclusions

In this work, the solubility of gaseous hydrocarbons in ILs was predicted using DBN, ELM, Boost-SVR, and MARS models. In this regard, the thermodynamic properties and chemical substructures of gaseous hydrocarbons and ILs along with temperature and pressure have been used in distinct methods to predict the solubility of gaseous hydrocarbons in ILs. The findings of this study give the following conclusions:

- In the method based on thermodynamic properties, the DBN model with RMSE and R^2^ values of 0.0054 and 0.9961, respectively, has the most accurate estimates.
- In the method based on chemical structure, temperature, and pressure, the DBN model with RMSE and R^2^ values of 0.0065 and 0.9943, respectively, has the best performance.
- Comparison of the results of smart models with equations of state shows that the accuracy of the former is much higher than the latter.
- The study of the effect of different factors on the solubility of gaseous hydrocarbons shows that increasing the pressure and molecular weight of hydrocarbons increases the solubility, while increasing the temperature decreases the solubility of gaseous hydrocarbons in ILs.
- Investigation of the effect of the type of anion and cation in the structure of ILs shows that the following order have been established for the solubility of the studied systems:
  Order of the solubility of C_2_H_6_ in ILs based on the anion type: [BMIM][PF_6_] < [BMIM][TF_2_N] < [BMIM][eFAP]
  Order of the solubility of CH_4_ in ILs based on the cation type: [tes][TF_2_N] < [bmpip][TF_2_N] < [cprop][TF_2_N] < [toa][TF_2_N]

## Data Availability

All the data have been collected from literature. We cited all the references of the data in the manuscript. However, the data will be available from the corresponding author on reasonable request.

## Abbreviations

ELM: Extreme learning machine
DBN: Deep belief network
B-SVR: Boosting support vector regression
MARS: Multivariate adaptive regression splines
RBM: Restricted Boltzmann machine
PR: Peng–Robinson
SRK: Soave–Redlich–Kwong
RK: Redlich–Kwong
ZJ: Zudkevitch–Joffe
EOS: Equation of state
RMSE: Root mean squared error
R^2^: Coefficient of determination
